# Comprehensive Outline of Whole Exome Sequencing Data Analysis Tools Available in Clinical Oncology

**DOI:** 10.3390/cancers11111725

**Published:** 2019-11-04

**Authors:** Áron Bartha, Balázs Győrffy

**Affiliations:** 1 Department of Bioinformatics and 2nd Department of Pediatrics, Semmelweis University, H-1094 Budapest, Hungary; bartha.aron@med.semmelweis-univ.hu; 2 TTK Cancer Biomarker Research Group, Institute of Enzymology, Magyar tudósokkörútja 2., H-1117 Budapest, Hungary

**Keywords:** whole exome sequencing, cancer, bioinformatics

## Abstract

Whole exome sequencing (WES) enables the analysis of all protein coding sequences in the human genome. This technology enables the investigation of cancer-related genetic aberrations that are predominantly located in the exonic regions. WES delivers high-throughput results at a reasonable price. Here, we review analysis tools enabling utilization of WES data in clinical and research settings. Technically, WES initially allows the detection of single nucleotide variants (SNVs) and copy number variations (CNVs), and data obtained through these methods can be combined and further utilized. Variant calling algorithms for SNVs range from standalone tools to machine learning-based combined pipelines. Tools for CNV detection compare the number of reads aligned to a dedicated segment. Both SNVs and CNVs help to identify mutations resulting in pharmacologically druggable alterations. The identification of homologous recombination deficiency enables the use of PARP inhibitors. Determining microsatellite instability and tumor mutation burden helps to select patients eligible for immunotherapy. To pave the way for clinical applications, we have to recognize some limitations of WES, including its restricted ability to detect CNVs, low coverage compared to targeted sequencing, and the missing consensus regarding references and minimal application requirements. Recently, Galaxy became the leading platform in non-command line-based WES data processing. The maturation of next-generation sequencing is reinforced by Food and Drug Administration (FDA)-approved methods for cancer screening, detection, and follow-up. WES is on the verge of becoming an affordable and sufficiently evolved technology for everyday clinical use.

## 1. Introduction

In the last decade, the price of genome sequencing has shrunk significantly, most of the work has become automated, and preparation guidelines have evolved. Due to these achievements, sequencing a whole genome has become a readily available possibility. Sequencing only targeting regions or the exome, however, implies a significantly smaller financial burden. In whole exome sequencing (WES), we primarily target specific fragments of the genome, the protein-coding part, and we therefore are able to identify genetic variants that will affect proteins. Since most of the known disease-causing mutations fall into this category, exome sequencing is a method that significantly reduces sequencing costs and therefore represents a clinically feasible approach for patient diagnostics. In this paper, we provide a summary of bioinformatic methods addressing the detection of the most frequent genetic aberrations influencing the development and progression of cancer.

Cancer is characterized by a set of essential steps that each renegade cell has to master before it can evolve to cancer [1]. The multitude of experimental methods that are at hand to investigate these cancer hallmarks have been systematically reviewed recently [2]. Whole exome sequencing provides a versatile tool to simultaneously monitor multiple different genomic changes in the tumor tissue. Mutations in both coding and noncoding DNA sequence regions have proven to be influential in the development of cancer [3,4]. Nucleic acid changes in the exome can result in amino acid changes in protein sequences. Amino acid changes lead to weakened activity of tumor suppressors, such as APC in colorectal cancer, VHL in renal cell cancer, or BRCA in breast cancer [5,6,7]. Copy number changes in cell cycle regulators, such as TP53 and RB1 [8], as well as malfunctions in repair mechanisms including the homologous recombination and DNA mismatch repair systems, predispose cells to cancer development. The activity of these repair systems can be monitored by measuring tumor mutational burden or microsatellite instability [9,10].

## 2. First Steps of Whole Exome Sequencing

At present, there are two main categories of next-generation sequencing (NGS) methods, consisting of DNA amplification-based sequencing (Illumina, Ion Torrent) and single molecule real-time sequencing (Pacific Biosciences, Oxford Nanopore). The investigated tissue samples can be freshly frozen, formalin-fixed and paraffin-embedded (FFPE), or liquid-based (blood sample); typically, each of these samples has its own isolation kits.

**Figure 1:**
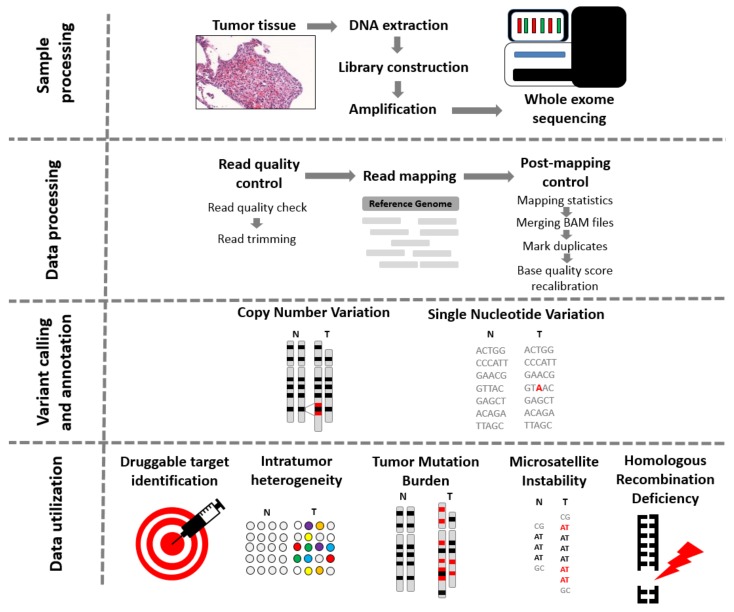
From tissue to data—steps of whole exome sequencing. Tissue preprocessing starts with the identification of tumor regions by an experienced pathologist, followed by DNA extraction, library construction, and amplification. Data procession commences with the quality check of reads. If the quality of trimmed reads is sufficient, the alignment of the reads to a reference genome is launched. When Binary Alignment Map (BAM) files are processed, the calling of single nucleotide variants, insertions and deletions, and copy number variants comes next, using one or more of the numerous existing algorithms. The data can be further utilized to detect microsatellite instability status, intratumor heterogeneity, tumor mutational burden, and homologous recombination deficiency.

A critical initial step of NGS is adequate pathological examination, as a properly selected and dissected tissue sample is a necessity for any further investigation [11]. Samples should contain a sufficient proportion of tumor cells to differentiate germline and somatic mutations. DNA from an adjacent normal tissue or from a blood sample is needed to identify all germ-line mutations. DNA quality deteriorates with time and after FFPE conservation, which has a degrading effect on the DNA. As the fragmentation of the DNA increases, the genome assembly following sequencing becomes more challenging [12]. During library construction, the exons are captured after an initial fragmentation step. Exome capture can be microarray-based or magnetic-bead based. In this second case, specific probes are hybridized to the sample, which are then pulled out using the magnetic beads. Then, the intronic sequences are discarded, and sequencing is performed using all the exonic sequences. The magnetic-bead-based capture methods are more widespread due to their simplicity [13]. To reach sufficient depth of coverage, properly capturing the targeted regions is necessary. Overall, currently used technologies deliver high efficiency [14]. Actual sequencing comes following exome capture and PCR amplification. The overall process of WES, including data processing and utilization, is summarized in Figure 1.

Usually, the data processing part starts with quality control and trimming at which low-quality reads are removed. This step is followed by the alignment of reads to a chosen reference genome followed by a second quality check step and removal of the duplicate reads. After these data processing steps, the variant calling splits, and at this point, a plethora of tools are available, depending on the clinical question one is attempting to answer.

## 3. Short Nucleotide Variants

**Figure 2:**
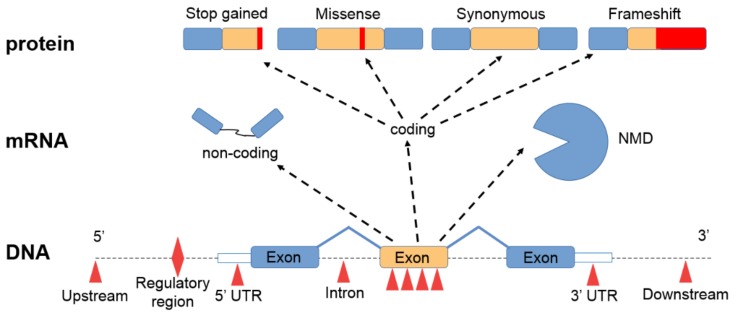
Effects of sequence alterations. Sequence variants in regulatory regions can activate or inhibit transcription. Mutations in exons result in an altered mRNA. Repair mechanisms, such as nonsense-mediated mRNA decay (NMD), can eliminate such abnormal mRNAs. As a result, missense mutations cause amino acid changes, while synonymous mutations result in the original amino acid sequence. Premature stop codons result in terminated amino acid sequences. Base insertions or deletions lead to frameshift mutations resulting in completely different proteins.

Whole exome sequencing is capable of delivering information for all protein-coding regions of the genome, which makes it a useful tool to identify germline and somatic mutations from a tumor sample (Figure 2). Compared to targeted sequencing, WES has the advantage of being able to elucidate the whole exome profile of a sample and to provide information on those low-frequency mutations that can collectively ground a complex phenotypic appearance [15]. Single nucleotide variants are able to increase the expression of key druggable targets, as has been suggested in lung [16], breast [17], colon [18], and gastric cancer [19].

**Figure 3:**
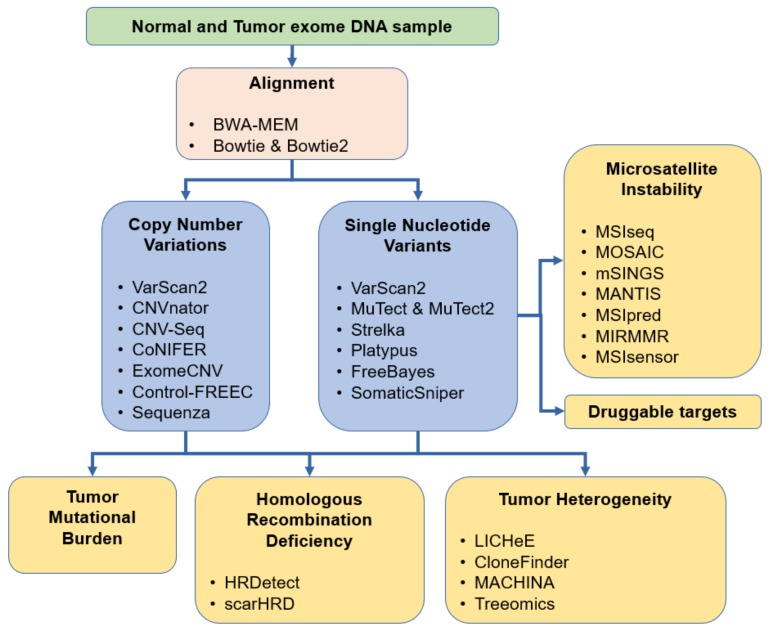
Overview of the most common methods for aberration detection useful in cancer diagnostics.

Accurate variant calling is a crucial component in the identification of such short variants. Currently, the most common variant caller tools in use include MuTect [20], VarScan2 [21], SomaticSniper [22], Strelka [23], and FreeBayes [24]. In addition, several clinical studies used a combination of these applications for variant calling [25,26,27,28,29,30,31,32,33,34,35]. A comprehensive list of all available tools is presented in Table 1, and the most common tools are presented in Figure 3.

According to a comparative analysis [57], selection of the right variant caller algorithm depends on the interest of variants. Some tools excel when dealing with low-coverage data (SomaticSniper [22], FaSD-somatic [54], and SNVSniffer [52]), while others perform better in regard to analyze low-frequency variants from high-coverage data (Strelka [23], MuTect [20], LoFreq [37], EBCall [41], deepSNV [45], LoLoPicker [56], and MuSE [42]). Other investigations also supported the approach of using specific variant callers: VarScan identified more high-quality single nucleotide variants (SNVs), while MuTect showed better performance in low-quality detection; therefore, the combinational usage of these can provide improved accuracy [58]. Examination of data from five breast cancer patients with nine variant caller algorithms affirmed the discrepant effect of coverage variability on the results [59]. Comparison of the four most frequently used applications (MuTect2, Strelka, VarsScan2, and SomaticSniper) lead to comparable results [60]. Each caller delivered a divergent outcome, although MuTect2 and Strelka outperformed VarScan and SomaticSniper in some cases. At the end, the authors conclude that the combination of tools could increase performance but with the sacrifice of a vast amount of detected calls [60]. Similar conclusions of complementary algorithms were drawn in another study evaluating four variant callers using whole exome sequencing and simulated data [61]. These researchers also noted differences based on different aligner tools. A further study also underlined the importance of the adequate mixture of aligner and variant caller selection and recommended the combination of the BWA-MEM aligner and SAMtools for SNP calling and the BWA-MEM GATK-Haplotype caller for indel detection [62]. It is important to note that in most comparative studies, the authors used the default settings of the tools; thus, for several methods, the performance might be improved by fine tuning and customization of filters.

## 4. Integrated Tools

Overall, different algorithms produce divergent output results. The utilization of combined pipelines can successfully filter the false positive hits and provide a platform for the customization of variant calling pipelines for the designated research objective. Such applications developed to deliver consensus Variant Call Format (VCF) files include VCFtools [63] NGS-pipe [64], VariantTools [65], vcfr [66], and myVCF [67]. These tools are notably useful when one aims to build pipelines that analyze VCF files generated in other tools (listed in the previous chapter). Other algorithms, such as Cake, can use BAM files as inputs. Cake runs all the variant caller tools separately and then unites the SNVs confirmed by at least two of the caller tools. Cake also offers numerous postprocessing filtering options [68]. Isma, an R package for the integrative analysis of mutations detected by multiple pipelines, provides a common platform for Strelka, MuTect/MuTect 2, MuSE, SomaticSniper, and VarScan2. Isma provides qualification for the used calling algorithms and highlights outlier results [69].

Using machine learning methods might further improve the specificity, sensitivity and comparability of these applications. BAYSIC integrates, among others, FreeBayes, SamTools, and GATK, and it can accept input from any variant caller algorithm [48]. SomaticSeq merges five algorithms (MuTect, VarScan2, SomaticSniper, JointSNVMix2, and VarDict), providing another machine learning-based ensembled application for SNV and indel identification [49]. SMuRF is another machine learning-based pipeline combining MuTect2, Freebayes, VarDict, and VarScan. SMuRF had the advantage of faster computing speed than other machine learning tools. While SMuRF outperformed several methods, it showed slightly poorer results than SomaticSeq; however, the time needed for SMuRF to compute the results was unsubstantial compared to SomaticSeq (10 min vs. 24 h) [70]. NeoMutate, a recently developed framework, also has the advantages of a mixture of separate tools and a machine learning-based perspective [71].

The application of machine learning ensemble methods has become increasingly accepted and shows a possible path for the development of future variant calling methods. However, currently implemented tools have an important drawback, as their sensitivity depends on that of the included algorithms.

## 5. Galaxy—An Open Source, Web-Based Platform

To use the applications discussed above, one has to possess advanced or at least intermediate programing skills, not to mention that many of these algorithms require different programming languages. Numerous user-friendly platforms have been established in the past years to overcome this obstacle. Generally, these are capable to give a platform in which users can build workflows made of genomic analysis tools. Researchers can use local workflow management systems like Taverna [72] or KNIME [73]. However, computing power is limited by the performance of the local computer. Cloud computing can serve as a possible solution for this issue [74]. Platforms like Cancer Genomics Cloud (CGC) [75], GenePattern [76], or Galaxy [77] are becoming more and more popular amongst scientists. Additional platforms available are listed in Table 2. Of these tools, Galaxy is the most widespread, due to the wide range of tools included and free availability. Users can utilize publicly available Galaxy servers or can set up their own private server.

When setting up a private server, one can include any of more than 5500 tools and algorithms from the Galaxy toolshed, which serves as an “AppStore” of applications [80]. However, establishing a private server requires constant maintenance and a skilled system administrator. Using a publicly available server, on the other hand, requires only a registration to the designated server, and the leading Galaxy servers already contain most commonly used tools. In addition to accessible research, Galaxy also has two additional important advantages: it makes it easier to reproduce analyses and provides a platform for users to communicate.

In regard to variant calling, Galaxy ToolShed provides numerous algorithms. The Galaxy training materials suggest a few recommended tools: VarScan for the identification of germline and somatic variants from tumor-normal sample pairs and FreeBayes for germ line variant calling.

As the clinical significance of variant caller methods expands, demands are increasing to solve specific problems. These problems include the detection of low-frequency variants—one possible solution could be utilization of unique molecule identifiers—and the accommodation of non-Illumina platforms. The perpetual improvement of the algorithmic tools is foreseeable if they want to compete with deep learning algorithms [57]. On the other hand, it is important to note that even the most well-established pipelines can be inefficient if the quality of utilized data is poor, e.g., inadequate exome capture, low coverage or modest sequencing quality [62].

## 6. Copy Number Variations

Copy number variations (CNVs) are structural changes of DNA, sized between a couple of hundred base changes and amplification or deletion of millions of base pairs [81]. The clinical relevance of CNVs in oncology has risen in the past several years, and CNVs have been indicated to be important in several types of cancer, such as adenomatous polyposis coli, familiar breast cancer, and ovarian cancer [8].

The clinically used gold standards for CNV detection are array Comparative Genome Hybridization (aCGH), Fluorescent In Situ Hybridization (FISH), and qPCR [82]. Current Food and Drug Administration (FDA)-approved methods for CNV detection are mainly FISH-based such as the “Dako TOP2A FISH PharmDX kit” for the detection of Topoisomerase 2-alpha aberrations or targeted sequencing based on the “FoundationOneCDx” NGS panel, which is capable of measuring the copy number changes in 324 genes. Each of the gold standard techniques is relatively inexpensive and provides reliable clinical data. Nonetheless, the opportunity to use sequencing can provide a robust amount of additional data with versatile further utility. Using whole genome sequencing (WGS) data for CNV detection has already been demonstrated to be useful [83]. However, due to financial issues, WGS is unlikely to become a clinical tool in the near future. WES, on the other hand, is a more affordable option to identify CNV changes.

Currently, dozens of algorithms and pipelines exist to detect CNVs from WES data; we have summarized these in Table 3, and the most common tools are listed in Figure 3. Most of the algorithms are based on the Read Depth approach, and they attempt to measure the CNV changes based on the number of reads aligned to a dedicated segment [84]. Although these algorithms can be relatively precise, normalization problems and other biases present as limitations of NGS technology. These limitations include contamination with normal cells, multiple types of clones among one sample and other experimental noises [85]. Only a few of the methods are capable of detecting CNV from cancer data, and substantial discrepancies can be observed when paralleling these tools. Although several studies have been conducted to compare these applications, only a few have focused on patients suffering from cancer as the study population.

Zare et. al. examined five algorithms on tumorous samples and concluded that some applications have achieved relatively good results on specificity and sensitivity [108]. In particular, ExomeCNV [90] showed high specificity and sensitivity with a moderate false discovery rate. SAAS-CNV [106] might be a useful tool for CNV detection; however, the specificity and sensitivity of the algorithm are inferior compared to the array methods [109].

Regarding overall specificity and sensitivity using simulated data [110], ADTEx [99] produced the best results followed by ControlFREEC [89], VarScan2 and ExomeCNV, but ExomeCNV and VarScan2 missed several homozygous deletions. Using breast cancer data in the same comparative study, ExomeCNV [90] showed the best results, while it produced moderate concordance with SNP arrays. Overall, ControlFREEC presented the best algorithm due to the balanced performance on both simulated and cancer data [110].

Based on the study examining six methods (ADTEx, CONTRA [95], ControlFREEC, EXCAVATOR, ExomeCNV, and VarScan2), these can identify homozygous deletions or large gains from WES data, but heterozygous deletions or low-level amplifications cannot be detected with sufficient consistency [111]. The results provided by ADTEx and EXCAVATOR were the most reliable [111].

Taken together, all the cited studies compare algorithms that were designed for somatic CNV detection from cancer-related data, and each came to a similar conclusion. At present, neither sensitivity nor specificity is precise enough to compete with the existing non-WES methods. Furthermore, multiple studies highlighted that using these algorithms on stimulated data shows better performance than on patient data, which indicates that the tools are not sufficiently fine-tuned to address tumor complexity, although some of them, such as ADTEx and ExomeCNV, have a built-in tool to tackle this issue.

Each application has different strengths and weaknesses; for instance, ADTEx can detect medium-sized CNVs, while EXCAVATOR is suitable for the identification of larger CNVs. Similar to SNVs, merging, fine tuning and recalibration of these tools could be a means of improving the specificity and sensitivity [112,113]. It is important to mention, however, that these discrepancies are not specific to somatic mutation detection, as similar issues appeared in germline mutation-based comparison [84].

Dealing with NGS data demands well-trained bioinformaticians because most of the algorithms can only be used in command line-based platforms. The availability of the aforementioned applications in Galaxy is slightly limited—to date, VarScan2 and a CNV caller part of the bcftools package are available in the basic Galaxy setup. Several further algorithms can be installed in the case of a private Galaxy server.

## 7. Homologous Recombination Deficiency

DNA double-strand breaks are one of the most mutagenic forms of DNA damage [114,115]. Cells have developed multiple solutions to confront these effects, such as homologous recombination and nonhomologous end-joining [116]. Germline mutations of the BRCA genes have been described as reliable markers to identify homologous recombination deficiency (HRD). Currently, one FDA-approved clinical tool is available to detect germline BRCA mutations, the BRACAnalysisCDx platform (Myriad Genetics; Salt Lake City, UT, USA), which is used to identify BRCA status in patients with ovarian cancer. The presence of a BRCA mutation enables treatment with a PARP inhibitor. PARP repairs single strand breaks, and the loss of both double-strand and single-strand break repair renders the tumor highly vulnerable to chemotherapy.

HRDetect is a WGS-based method to identify the presence of homologous recombination repair mechanism mutations; this tool has proven to be effective and reliable regardless of germline and somatic mutation or tissue type. However, using this tool on WES data revealed a considerable decrease in the detection sensitivity [117]. Another recent WES-based tool promises comparable results with SNP array examinations based on genomic scar analysis and might be a useful tool to detect BRCA status [118]. Since HRD detection mainly focuses on BRCA status, we currently have a lack of application capable of measuring overall HRD status involving all related genetic aberrations. Meanwhile, several other genes have also been shown to play important roles in HRD [119]. An improved future WES-based algorithm could enable the simultaneous investigation of all involved genes.

## 8. Response to Immunotherapy

Immune checkpoint inhibitors and immunomodulatory agents have become standard treatments for solid tumors, including renal-cell carcinoma, melanoma and NSCLC [120]. The number of mutations per coding sequence in the tumor genome is a reliable predictive biomarker of immunotherapy response [121]. At present, the application of WES to detect tumor mutational burden (TMB) is a widely accepted gold standard. In addition, multiple targeted panels have also been accepted as targeted sequencing show comparable results in the detection of TMB status as exome sequencing [122].

Although TMB bears strong potential as a predictive biomarker, there is a lack of unambiguous consensus on the correct determination, definition, and cut-off values. The Friends of Cancer Research established a working group to create a universal reference and harmonize these methods to address this issue [123]. Because of the lack of solid guidelines, various studies have used numerous methods and computational techniques for TMB status determination. We evaluated eleven phase II and III clinical studies, and MuTect was the most frequently used tool for somatic variant detection, while the applications applied for InDel detection showed a wide variety [25,26,27,28,29,30,31,32,33,34,35]. A significant set of publications use the pipeline proposed by the Genome Analysis Toolkit—supplementing it with additional tools—which recommends GATK-Mutect2, which is based on MuTect and the GATK-HaplotypeCaller.

Another concept recently gaining attention is the examination of mutational signatures. Mutations in cancer can originate in different mutagenic effects or defects in repair mechanisms. Each genetic aberration has its unique mutational signature which can include base substitutions, small insertions and deletions, CNV changes, or genomic rearrangements [124]. As the quantity of explored signatures is growing, a systematic and curated archive of genetic patterns is needed. The Catalogue of Somatic Mutations in Cancer (COSMIC) provides such a repository for mutational signatures and specific summary vignettes. Deciphering characteristic mutational patterns in a chosen cancer type requires bioinformatic analysis as well. Currently, there are several algorithms designed for mutational landscape identification, such as SigProfiler [125], deconstrutSigs [126], and mutationalPatterns [127]. HRDetect, a tool developed as a kind of mutational signature detecting algorithm designed for the identification of homologue recombination repair deficiency, has been already discussed in a separate paragraph. Accepted analysis standards for these methods are still missing [128]. Clinical cancer diagnostics might benefit from the application of mutational signature detection, as aberration patterns can be useful for targeted treatment selection [129]. 

A different predictive biomarker for immune modulatory response is the evaluation of Microsatellite Instability (MSI). From the time when the FDA approved pembrolizumab for the treatment of adult and pediatric microsatellite instability high (MSI-H) or mismatch repair-deficient (dMMR) solid tumors, MSI detection gathered significant clinical attention [29]. A recent study suggests that impaired mismatch repair activity might result in higher mutational burden resulting in augmented response to immunomodulatory agents [130]. The currently existing method for MSI detection, known as the combination of PCR with fluorescent primers and capillary electrophoresis, is becoming obsolete with the introduction of WES and targeted gene panel sequencing [131].

At present, the number of applications for MSI identification from exome sequencing data is not as high as the number of those for CNV or short variant detection. Comparing some of these tools in six cancer types revealed that MANTIS produces better sensitivity and specificity than MSIsensor and mSINGS [132]. MSIseq show results comparable to MSISensor and mSINGS, while the MSIseq R package runs much faster than the two other [133]. MSIseq and MSIpred have the advantage that these algorithms can measure MSI from tumor data only. Based on data comparison using TCGA data, MSIpred exhibited higher accuracy and sensitivity than MSIseq [134]. MIRMMR also displayed similarities in accuracy and sensitivity with MSIsensor and mSINGS [135]. A recently implemented tool based on the examination of 5930 tumor exomes across 18 cancer types, called MOSAIC, produced remarkable sensitivity and specificity [136]. Overall, out of the seven algorithms available, MOSAIC has the strongest and most well-established analytical background, while MSIpred shows better performance than others with the advantage that it can operate without a normal reference sample.

Unfortunately, no specific tool has been developed for MSI detection from exome sequencing data for those who have less experience in command line coding. This finding indicates that the Galaxy platform is the only alternative.

Finally, predicting response to immunotherapy has an additional option as—according to a state-of-the-art paper—elevated DNA damage might be a possible biomarker of response [137].

## 9. Tumor Heterogeneity

Tumor heterogeneity stands for diversity within one tumor population, where several different populations coexist. These cancerous populations coexist with normal cells and infiltrating immune-related cells in a special microenvironment. The subclonal populations can cooperatively evolve and are even capable of adapting to altered circumstances, including the emergence of therapy-resistant clones following systemic anticancer treatments [138]. Currently, there is no broadly accepted consensus method for the estimation of tumor heterogeneity. Identification of the clonal subpopulations is possible by all three sequencing methods—WGS, WES, and targeted panel—and by single-cell approaches.

A widely accepted way to measure tumor diversity is the use of WES to measure the genetic heterogeneity of a tumor sample by counting Shannon’s diversity index of the estimated SNVs [139]. The determination of tumor clonality and evolutionary background from bulk sequencing data is a multistep process. This method begins with the cancer cell fraction estimation, then the identification of tumor subclones followed by the construction of a phylogenic tree based on the distribution of somatic variants and/or CNV status. Finally, temporal differentiation can assist in distinguishing between passenger and driver mutations [140].

In addition to the aforementioned approach, numerous algorithms have been developed to illuminate subclone phylogenesis. Unfortunately, due to the scarcity of comparative studies, we have only limited guidance on proper algorithm selection at this time. In a study of nine methods, LICHeE and CloneFinder produced decent accuracy compared to the others [141]. In a recent comparative study currently available in a preprint server only, the authors examined seven clonality prediction methods. CloneFinder, MACHINA, Treeomics, and LICHeE showed the best performance, but it is important to mention that none of the applications showed impaired overall performance on all the stimulated datasets [142].

Overall, the examination of tumor heterogeneity by NGS-based methods has a limited history, and because of this reason, many of the currently existing methods require further fine-tuning. In vitro experiments might serve as guidance for adequate algorithm calibration and could provide further information on the detection threshold and coverage cut-off value selection. Recently, we have shown that cellular movement can also lead to a significant technical bias when using NGS to determine the clonal composition of a tumor [143]. With the technical development of both bulk sequencing and single cell methods, we will soon be able to confidently obtain an accurate picture of a cancer population in its complete heterogeneity.

## 10. Discussion

The first U.S. Food and Drug Administration (FDA) approval for NGS technology was issued in 2013, and a few years later, the approval of the first tests for diagnostic and screening was granted. We provide an overview of NGS-based tests approved for somatic or germline mutation detection in Table 4.

We are now in the big data era borne by the vast amount of data delivered by new sequencing methods. Deciphering this information requires complex bioinformatical analytical tools. At the same time, we have to account for the unquestionable weaknesses of exome sequencing [144]. These disadvantages include the limited power to detect structural gene fusions and the limited ability to delineate tumor purity and differentiate from normal cell contamination. The previously discussed machine learning algorithms in short variant detection can improve the accuracy of TMB and MSI detection, as punctual short variant identification is a crucial part of both. Improved detection of copy number changes can lead to more accurate HRD and tumor heterogeneity analysis [145].

The final outcome of our paper is that, due to discrepancies amongst tools used during sample preparation and data preprocessing and processing, it is almost impossible to define a gold standard guideline of the most handy algorithms. Of note, anyone can customize the selected algorithms specifically for their own experiment rather than using it on default settings.

The clinical significance of NGS-based methods is consistently expanding. Although discrepancies can be observed among the currently available tools, the continuous fine-tuning and the merged utilization of these applications paves the way for clinically reliable applications in the coming years. Overall, WES is emerging as a future “Swiss army knife” of cancer genome profiling. After as bioinformatic processes have evolved to trustworthy pipelines, WES will be an affordable and mature technology for everyday clinical use.

## Figures and Tables

**Table 1:** Bioinformatic methods available for single nucleotide variant calling. Tools marked with an asterisk (*) are suitable for both whole genome sequencing (WGS) and whole exome sequencing (WES) data analysis.

Name	Published	Cited in 2018	Control Needed	InDel detection	Contamination Correction	Trained on Cancer Data	Environment	Ref
Varscan2	2012	2229	+	+	−	+	Java, Perl, R, Galaxy	[21]
MuTect2 *	2013	2005	+	−	+	+	Java, R	[20]
FreeBayes	2012	1121	−	+	−	+	C, C++, Galaxy	[24]
Strelka *	2012	759	+	+	−	+	C++, Perl	[23]
Platypus *	2014	462	−	+	−	+	C, Cython, Python	[36]
SomaticSniper *	2012	373	+	−	−	+	C, Galaxy	[22]
LoFreq *	2012	349	−	+	+	+	Python	[37]
VarDict *	2016	171	−	+	−	+	Perl	[38]
JointSNVMix *	2012	160	+	−	−	+	C, C++, Python, Galaxy	[39]
MutationSeq *	2012	108	+	−	−	+	C++, Python	[40]
EBCall *	2013	85	+	+	−	+	C++, Perl, R, Shell	[41]
MuSE *	2016	65	+	−	+	+	C, C++	[42]
RADIA	2014	53	+	−	+	+	Python	[43]
Virmid	2013	49	+	−	+	+	Java	[44]
deepSNV *	2014	47	+	−	−	+	R	[45]
Shimmer *	2013	45	+	−	+	+	C, Perl, R	[46]
qSNP *	2013	40	+	−	+	−	Java	[47]
BAYSIC	2014	39	+	−	−	+	R	[48]
SomaticSeq *	2015	38	+	+	−	+	Python, R	[49]
CaVEMan *	2016	31	+	−	+	+	C	[50]
SNooPer *	2016	26	−	+	+	+	Perl	[51]
SNVSniffer *	2016	17	−	+	−	+	C++	[52]
HapMuC	2014	15	−	+	−	+	C++, Python, Ruby	[53]
FaSD-somatic	2014	13	−	−	−	+	C, C++	[54]
LocHap *	2016	8	+	+	+	+	g++ complier, GNU Make	[55]
LoLoPicker *	2017	6	+	−	+	+	Python	[56]

**Table 2:** Platforms available for bioinformatic analysis.

Name	Description	Year	Citation	License	System type	Ref.
Galaxy	Open-source web-platform with several analysis tools	2005	1977	free	cloud-based	[77]
GenePattern	Workflow management system, provides access to multiple genomic analysis tools	2006	1573	free	cloud-based	[76]
KNIME	Software enabling creation, analysis, and visualization of data	2008	1476	free	local installation needed	[73]
UGENE	Workflow management system installed on a local computer	2012	876	free	local installation needed	[78]
Taverna	Open source software tool for designing and executing workflows	2013	643	free	local installation needed	[72]
Cancer Genomics Cloud	Provides access to data, tools, and computing resources	2017	32	commercial	cloud-based	[75]
SciApps	Platform for building, running, and sharing scientific workflows	2018	5	free	cloud-based	[79]
Terra	Bioinformatic workspace, including a repository of public best practices, methods, and public data sets	−	−	commercial	cloud-based	−

**Table 3:** Computational methods available for copy number variation estimation from whole exome sequencing data. Tools marked with an asterisk are suitable for both WGS and WES data analysis.

Name	Published	Control Needed	Contamination Correction	GC-Content Correction	Trained on Cancer Data	Cited in 2018	Environment	Ref.
Varscan2	2012	+	−	−	+	2229	Java, Perl, R, Galaxy	[21]
CNVnator	2011	+	−	+	−	767	C++	[86]
CNV-Seq	2009	+	−	−	−	463	Perl, R	[87]
CoNIFER	2012	−	+	−	−	378	Python	[88]
Control-FREEC *	2012	−	+	+	+	342	C, C++, R	[89]
ExomeCNV	2011	+	+	−	+	338	R	[90]
XHMM	2012	−	+	+	+	322	C++	[91]
ExomeDepth	2012	+	−	+	−	264	R	[92]
cn.MOPS	2012	−	+	+	−	249	R	[93]
Cnvkit *	2016	+	+	+	+	219	Python, Galaxy	[94]
CONTRA	2012	−	−	+	−	194	Python, R	[95]
Sequenza *	2015	+	−	+	+	167	Python, R	[96]
EXCAVATOR	2013	+	+	+	+	155	Perl	[97]
CODEX	2015	−	+	+	+	72	R	[98]
ADTEx	2014	+	+	−	+	57	Python, R	[99]
Seqgene	2011	+	−	−	+	43	R	[100]
FishingCNV	2013	−	−	−	−	41	Java, R	[101]
HMZDelFinder	2017	−	−	−	−	33	R	[102]
ExoCNVTest	2012	+	−	−	−	27	Java, R	[103]
CLAMMS	2016	−	−	+	−	23	C	[104]
falcon	2015	+	+	−	+	22	C	[105]
saasCNV *	2015	+	+	−	+	17	R	[106]
WISExome	2017	−	−	−	−	1	C, C++	[107]

**Table 4:** Food and Drug Administration (FDA)-approved next-generation sequencing (NGS)-based methods suitable for cancer predisposition identification, cancer detection, or follow-up.

Tradename	Description	Year	Target	Tumor	Utility
Illumina MiSeqDX platform	High throughput DNA sequence analyzer	2013	-	-	technology
FoundationFocus CDxBRCA	NGS oncology panel, somatic or germline variant detection system	2016	BRCA	ovarian	diagnosis
MSK-IMPACT	NGS-based tumor profiling test	2017	468 genes	various	predisposition, diagnosis
FoundationOne CDx	NGS oncology panel, somatic or germline variant detection system	2017	324 genes	various	predisposition, diagnosis
Oncomine Dx Target Test	NGS oncology panel, somatic or germline variant detection system	2017	24 genes	lung	diagnosis
Praxis Extended RAS Panel	NGS oncology panel, somatic or germline variant detection system	2017	RAS	colon	diagnosis
Adaptive Biotechnologies clonoSEQ	DNA-based test for minimal residual disease for hematologic malignancies	2018	BCL1, BCL2	leukemia, myeloma	follow-up

## References

[1] Hanahan D., Weinberg R.A. (2011). Hallmarks of cancer: The next generation. Cell.

[2] Menyhart O., Harami-Papp H., Sukumar S., Schafer R., Magnani L., de Barrios O., Gyorffy B. (2016). Guidelines for the selection of functional assays to evaluate the hallmarks of cancer. Biochim. Biophys. Acta.

[3] Schaub M.A., Boyle A.P., Kundaje A., Batzoglou S., Snyder M. (2012). Linking disease associations with regulatory information in the human genome. Genome Res..

[4] Li G., Pan T., Guo D., Li L.C. (2014). Regulatory Variants and Disease: The E-Cadherin -160C/A SNP as an Example. Mol. Biol. Int..

[5] Minde D.P., Anvarian Z., Rudiger S.G., Maurice M.M. (2011). Messing up disorder: How do missense mutations in the tumor suppressor protein APC lead to cancer?. Mol. Cancer.

[6] Gnarra J.R., Tory K., Weng Y., Schmidt L., Wei M.H., Li H., Latif F., Liu S., Chen F., Duh F.M. (1994). Mutations of the VHL tumour suppressor gene in renal carcinoma. Nat. Genet..

[7] Farmer H., McCabe N., Lord C.J., Tutt A.N., Johnson D.A., Richardson T.B., Santarosa M., Dillon K.J., Hickson I., Knights C. (2005). Targeting the DNA repair defect in BRCA mutant cells as a therapeutic strategy. Nature.

[8] Shlien A., Malkin D. (2009). Copy number variations and cancer. Genome Med..

[9] Torgovnick A., Schumacher B. (2015). DNA repair mechanisms in cancer development and therapy. Front. Genet..

[10] Luchini C., Bibeau F., Ligtenberg M.J.L., Singh N., Nottegar A., Bosse T., Miller R., Riaz N., Douillard J.Y., Andre F. (2019). ESMO recommendations on microsatellite instability testing for immunotherapy in cancer, and its relationship with PD-1/PD-L1 expression and tumour mutational burden: A systematic review-based approach. Ann. Oncol. Off. J. Eur. Soc. Med Oncol..

[11] Morlote D., Janowski K.M., Siniard R.C., Guo R.J., Winokur T., DeFrank G., Harada S. (2019). Effects of Improved DNA Integrity by Punch from Tissue Blocks as Compared to Pinpoint Extraction from Unstained Slides on Next-Generation Sequencing Quality Metrics. Am. J. Clin. Pathol..

[12] McDonough S.J., Bhagwate A., Sun Z., Wang C., Zschunke M., Gorman J.A., Kopp K.J., Cunningham J.M. (2019). Use of FFPE-derived DNA in next generation sequencing: DNA extraction methods. PLoS ONE.

[13] Warr A., Robert C., Hume D., Archibald A., Deeb N., Watson M. (2015). Exome Sequencing: Current and Future Perspectives. G3 Genes Genomes Genet..

[14] Chilamakuri C.S., Lorenz S., Madoui M.A., Vodak D., Sun J., Hovig E., Myklebost O., Meza-Zepeda L.A. (2014). Performance comparison of four exome capture systems for deep sequencing. BMC Genom..

[15] Pongor L., Kormos M., Hatzis C., Pusztai L., Szabo A., Gyorffy B. (2015). A genome-wide approach to link genotype to clinical outcome by utilizing next generation sequencing and gene chip data of 6697 breast cancer patients. Genome Med..

[16] Nagy A., Pongor L.S., Szabo A., Santarpia M., Gyorffy B. (2017). KRAS driven expression signature has prognostic power superior to mutation status in non-small cell lung cancer. Int. J. Cancer.

[17] Gyorffy B., Pongor L., Bottai G., Li X., Budczies J., Szabo A., Hatzis C., Pusztai L., Santarpia L. (2018). An integrative bioinformatics approach reveals coding and non-coding gene variants associated with gene expression profiles and outcome in breast cancer molecular subtypes. Br. J. Cancer.

[18] Menyhart O., Kakisaka T., Pongor L.S., Uetake H., Goel A., Gyorffy B. (2019). Uncovering Potential Therapeutic Targets in Colorectal Cancer by Deciphering Mutational Status and Expression of Druggable Oncogenes. Cancers.

[19] Menyhart O., Pongor L.S., Gyorffy B. (2018). Mutations Defining Patient Cohorts with Elevated PD-L1 Expression in Gastric Cancer. Front. Pharmacol..

[20] Cibulskis K., Lawrence M.S., Carter S.L., Sivachenko A., Jaffe D., Sougnez C., Gabriel S., Meyerson M., Lander E.S., Getz G. (2013). Sensitive detection of somatic point mutations in impure and heterogeneous cancer samples. Nat. Biotechnol..

[21] Koboldt D.C., Zhang Q., Larson D.E., Shen D., McLellan M.D., Lin L., Miller C.A., Mardis E.R., Ding L., Wilson R.K. (2012). VarScan 2: Somatic mutation and copy number alteration discovery in cancer by exome sequencing. Genome Res..

[22] Larson D.E., Harris C.C., Chen K., Koboldt D.C., Abbott T.E., Dooling D.J., Ley T.J., Mardis E.R., Wilson R.K., Ding L. (2012). SomaticSniper: Identification of somatic point mutations in whole genome sequencing data. Bioinformatics.

[23] Saunders C.T., Wong W.S., Swamy S., Becq J., Murray L.J., Cheetham R.K. (2012). Strelka: Accurate somatic small-variant calling from sequenced tumor-normal sample pairs. Bioinformatics.

[24] Erik Garrison G.M. (2012). Haplotype-based variant detection from short-read sequencing. arXiv.

[25] Carbone D.P., Reck M., Paz-Ares L., Creelan B., Horn L., Steins M., Felip E., van den Heuvel M.M., Ciuleanu T.E., Badin F. (2017). First-Line Nivolumab in Stage IV or Recurrent Non-Small-Cell Lung Cancer. N. Engl. J. Med..

[26] Cristescu R., Mogg R., Ayers M., Albright A., Murphy E., Yearley J., Sher X., Liu X.Q., Lu H., Nebozhyn M. (2018). Pan-tumor genomic biomarkers for PD-1 checkpoint blockade-based immunotherapy. Science.

[27] Hellmann M.D., Nathanson T., Rizvi H., Creelan B.C., Sanchez-Vega F., Ahuja A., Ni A., Novik J.B., Mangarin L.M.B., Abu-Akeel M. (2018). Genomic Features of Response to Combination Immunotherapy in Patients with Advanced Non-Small-Cell Lung Cancer. Cancer Cell.

[28] Hugo W., Zaretsky J.M., Sun L., Song C., Moreno B.H., Hu-Lieskovan S., Berent-Maoz B., Pang J., Chmielowski B., Cherry G. (2016). Genomic and Transcriptomic Features of Response to Anti-PD-1 Therapy in Metastatic Melanoma. Cell.

[29] Le D.T., Uram J.N., Wang H., Bartlett B.R., Kemberling H., Eyring A.D., Skora A.D., Luber B.S., Azad N.S., Laheru D. (2015). PD-1 Blockade in Tumors with Mismatch-Repair Deficiency. N. Engl. J. Med..

[30] Riaz N., Havel J.J., Makarov V., Desrichard A., Urba W.J., Sims J.S., Hodi F.S., Martin-Algarra S., Mandal R., Sharfman W.H. (2017). Tumor and Microenvironment Evolution during Immunotherapy with Nivolumab. Cell.

[31] Rizvi H., Sanchez-Vega F., La K., Chatila W., Jonsson P., Halpenny D., Plodkowski A., Long N., Sauter J.L., Rekhtman N. (2018). Molecular Determinants of Response to Anti-Programmed Cell Death (PD)-1 and Anti-Programmed Death-Ligand 1 (PD-L1) Blockade in Patients With Non-Small-Cell Lung Cancer Profiled With Targeted Next-Generation Sequencing. J. Clin. Oncol. Off. J. Am. Soc. Clin. Oncol..

[32] Rizvi N.A., Hellmann M.D., Snyder A., Kvistborg P., Makarov V., Havel J.J., Lee W., Yuan J., Wong P., Ho T.S. (2015). Cancer immunology. Mutational landscape determines sensitivity to PD-1 blockade in non-small cell lung cancer. Science.

[33] Snyder A., Makarov V., Merghoub T., Yuan J., Zaretsky J.M., Desrichard A., Walsh L.A., Postow M.A., Wong P., Ho T.S. (2014). Genetic basis for clinical response to CTLA-4 blockade in melanoma. N. Engl. J. Med..

[34] Snyder A., Nathanson T., Funt S.A., Ahuja A., Buros Novik J., Hellmann M.D., Chang E., Aksoy B.A., Al-Ahmadie H., Yusko E. (2017). Contribution of systemic and somatic factors to clinical response and resistance to PD-L1 blockade in urothelial cancer: An exploratory multi-omic analysis. PLoS Med..

[35] Van Allen E.M., Miao D., Schilling B., Shukla S.A., Blank C., Zimmer L., Sucker A., Hillen U., Foppen M.H.G., Goldinger S.M. (2015). Genomic correlates of response to CTLA-4 blockade in metastatic melanoma. Science.

[36] Rimmer A., Phan H., Mathieson I., Iqbal Z., Twigg S.R.F., Consortium W.G.S., Wilkie A.O.M., McVean G., Lunter G. (2014). Integrating mapping-, assembly- and haplotype-based approaches for calling variants in clinical sequencing applications. Nat. Genet..

[37] Wilm A., Aw P.P., Bertrand D., Yeo G.H., Ong S.H., Wong C.H., Khor C.C., Petric R., Hibberd M.L., Nagarajan N. (2012). LoFreq: A sequence-quality aware, ultra-sensitive variant caller for uncovering cell-population heterogeneity from high-throughput sequencing datasets. Nucleic Acids Res..

[38] Lai Z., Markovets A., Ahdesmaki M., Chapman B., Hofmann O., McEwen R., Johnson J., Dougherty B., Barrett J.C., Dry J.R. (2016). VarDict: A novel and versatile variant caller for next-generation sequencing in cancer research. Nucleic Acids Res..

[39] Roth A., Ding J., Morin R., Crisan A., Ha G., Giuliany R., Bashashati A., Hirst M., Turashvili G., Oloumi A. (2012). JointSNVMix: A probabilistic model for accurate detection of somatic mutations in normal/tumour paired next-generation sequencing data. Bioinformatics.

[40] Ding J., Bashashati A., Roth A., Oloumi A., Tse K., Zeng T., Haffari G., Hirst M., Marra M.A., Condon A. (2012). Feature-based classifiers for somatic mutation detection in tumour-normal paired sequencing data. Bioinformatics.

[41] Shiraishi Y., Sato Y., Chiba K., Okuno Y., Nagata Y., Yoshida K., Shiba N., Hayashi Y., Kume H., Homma Y. (2013). An empirical Bayesian framework for somatic mutation detection from cancer genome sequencing data. Nucleic Acids Res..

[42] Fan Y., Xi L., Hughes D.S., Zhang J., Zhang J., Futreal P.A., Wheeler D.A., Wang W. (2016). MuSE: Accounting for tumor heterogeneity using a sample-specific error model improves sensitivity and specificity in mutation calling from sequencing data. Genome Biol..

[43] Radenbaugh A.J., Ma S., Ewing A., Stuart J.M., Collisson E.A., Zhu J., Haussler D. (2014). RADIA: RNA and DNA integrated analysis for somatic mutation detection. PLoS ONE.

[44] Kim S., Jeong K., Bhutani K., Lee J., Patel A., Scott E., Nam H., Lee H., Gleeson J.G., Bafna V. (2013). Virmid: Accurate detection of somatic mutations with sample impurity inference. Genome Biol..

[45] Gerstung M., Papaemmanuil E., Campbell P.J. (2014). Subclonal variant calling with multiple samples and prior knowledge. Bioinformatics.

[46] Hansen N.F., Gartner J.J., Mei L., Samuels Y., Mullikin J.C. (2013). Shimmer: Detection of genetic alterations in tumors using next-generation sequence data. Bioinformatics.

[47] Kassahn K.S., Holmes O., Nones K., Patch A.M., Miller D.K., Christ A.N., Harliwong I., Bruxner T.J., Xu Q., Anderson M. (2013). Somatic point mutation calling in low cellularity tumors. PLoS ONE.

[48] Cantarel B.L., Weaver D., McNeill N., Zhang J., Mackey A.J., Reese J. (2014). BAYSIC: A Bayesian method for combining sets of genome variants with improved specificity and sensitivity. BMC Bioinform..

[49] Fang L.T., Afshar P.T., Chhibber A., Mohiyuddin M., Fan Y., Mu J.C., Gibeling G., Barr S., Asadi N.B., Gerstein M.B. (2015). An ensemble approach to accurately detect somatic mutations using SomaticSeq. Genome Biol..

[50] Jones D., Raine K.M., Davies H., Tarpey P.S., Butler A.P., Teague J.W., Nik-Zainal S., Campbell P.J. (2016). cgpCaVEManWrapper: Simple Execution of CaVEMan in Order to Detect Somatic Single Nucleotide Variants in NGS Data. Curr. Protoc. Bioinform..

[51] Spinella J.F., Mehanna P., Vidal R., Saillour V., Cassart P., Richer C., Ouimet M., Healy J., Sinnett D. (2016). SNooPer: A machine learning-based method for somatic variant identification from low-pass next-generation sequencing. BMC Genom..

[52] Liu Y., Loewer M., Aluru S., Schmidt B. (2016). SNVSniffer: An integrated caller for germline and somatic single-nucleotide and indel mutations. BMC Syst. Biol..

[53] Usuyama N., Shiraishi Y., Sato Y., Kume H., Homma Y., Ogawa S., Miyano S., Imoto S. (2014). HapMuC: Somatic mutation calling using heterozygous germ line variants near candidate mutations. Bioinformatics.

[54] Wang W., Wang P., Xu F., Luo R., Wong M.P., Lam T.W., Wang J. (2014). FaSD-somatic: A fast and accurate somatic SNV detection algorithm for cancer genome sequencing data. Bioinformatics.

[55] Sengupta S., Gulukota K., Zhu Y., Ober C., Naughton K., Wentworth-Sheilds W., Ji Y. (2016). Ultra-fast local-haplotype variant calling using paired-end DNA-sequencing data reveals somatic mosaicism in tumor and normal blood samples. Nucleic Acids Res..

[56] Carrot-Zhang J., Majewski J. (2017). LoLoPicker: Detecting low allelic-fraction variants from low-quality cancer samples. Oncotarget.

[57] Xu C. (2018). A review of somatic single nucleotide variant calling algorithms for next-generation sequencing data. Comput. Struct. Biotechnol. J..

[58] Liu Z.K., Shang Y.K., Chen Z.N., Bian H. (2017). A three-caller pipeline for variant analysis of cancer whole-exome sequencing data. Mol. Med. Rep..

[59] Kroigard A.B., Thomassen M., Laenkholm A.V., Kruse T.A., Larsen M.J. (2016). Evaluation of Nine Somatic Variant Callers for Detection of Somatic Mutations in Exome and Targeted Deep Sequencing Data. PLoS ONE.

[60] Cai L., Yuan W., Zhang Z., He L., Chou K.C. (2016). In-depth comparison of somatic point mutation callers based on different tumor next-generation sequencing depth data. Sci. Rep..

[61] Kumaran M., Subramanian U., Devarajan B. (2019). Performance assessment of variant calling pipelines using human whole exome sequencing and simulated data. BMC Bioinform..

[62] Hwang S., Kim E., Lee I., Marcotte E.M. (2015). Systematic comparison of variant calling pipelines using gold standard personal exome variants. Sci. Rep..

[63] Danecek P., Auton A., Abecasis G., Albers C.A., Banks E., DePristo M.A., Handsaker R.E., Lunter G., Marth G.T., Sherry S.T. (2011). The variant call format and VCFtools. Bioinformatics.

[64] Singer J., Ruscheweyh H.J., Hofmann A.L., Thurnherr T., Singer F., Toussaint N.C., Ng C.K.Y., Piscuoglio S., Beisel C., Christofori G. (2018). NGS-pipe: A flexible, easily extendable and highly configurable framework for NGS analysis. Bioinformatics.

[65] Lawrence M., Gentleman R. (2017). VariantTools: An extensible framework for developing and testing variant callers. Bioinformatics.

[66] Knaus B.J., Grunwald N.J. (2017). vcfr: A package to manipulate and visualize variant call format data in R. Mol. Ecol. Resour..

[67] Pietrelli A., Valenti L. (2017). myVCF: A desktop application for high-throughput mutations data management. Bioinformatics.

[68] Rashid M., Robles-Espinoza C.D., Rust A.G., Adams D.J. (2013). Cake: A bioinformatics pipeline for the integrated analysis of somatic variants in cancer genomes. Bioinformatics.

[69] Di Nanni N., Moscatelli M., Gnocchi M., Milanesi L., Mosca E. (2019). isma: An R package for the integrative analysis of mutations detected by multiple pipelines. BMC Bioinform..

[70] Huang W., Guo Y.A., Muthukumar K., Baruah P., Chang M.M., Skanderup A.J. (2019). SMuRF: Portable and accurate ensemble prediction of somatic mutations. Bioinformatics.

[71] Anzar I., Sverchkova A., Stratford R., Clancy T. (2019). NeoMutate: An ensemble machine learning framework for the prediction of somatic mutations in cancer. BMC Med. Genom..

[72] Wolstencroft K., Haines R., Fellows D., Williams A., Withers D., Owen S., Soiland-Reyes S., Dunlop I., Nenadic A., Fisher P. (2013). The Taverna workflow suite: Designing and executing workflows of Web Services on the desktop, web or in the cloud. Nucleic Acids Res..

[73] Berthold M.R., Cebron N., Dill F., Gabriel T.R., Kötter T., Meinl T., Ohl P., Sieb C., Thiel K., Wiswedel B. (2008). KNIME: The Konstanz Information Miner.

[74] Langmead B., Nellore A. (2018). Cloud computing for genomic data analysis and collaboration. Nat. Rev. Genet..

[75] Lau J.W., Lehnert E., Sethi A., Malhotra R., Kaushik G., Onder Z., Groves-Kirkby N., Mihajlovic A., DiGiovanna J., Srdic M. (2017). The Cancer Genomics Cloud: Collaborative, Reproducible, and Democratized-A New Paradigm in Large-Scale Computational Research. Cancer Res..

[76] Reich M., Liefeld T., Gould J., Lerner J., Tamayo P., Mesirov J.P. (2006). GenePattern 2.0. Nat. Genet..

[77] Giardine B., Riemer C., Hardison R.C., Burhans R., Elnitski L., Shah P., Zhang Y., Blankenberg D., Albert I., Taylor J. (2005). Galaxy: A platform for interactive large-scale genome analysis. Genome Res..

[78] Okonechnikov K., Golosova O., Fursov M., Ugene Team (2012). Unipro UGENE: A unified bioinformatics toolkit. Bioinformatics.

[79] Wang L., Lu Z., Van Buren P., Ware D. (2018). SciApps: A cloud-based platform for reproducible bioinformatics workflows. Bioinformatics.

[80] Afgan E., Baker D., Batut B., van den Beek M., Bouvier D., Cech M., Chilton J., Clements D., Coraor N., Gruning B.A. (2018). The Galaxy platform for accessible, reproducible and collaborative biomedical analyses: 2018 update. Nucleic Acids Res..

[81] Conrad D.F., Pinto D., Redon R., Feuk L., Gokcumen O., Zhang Y., Aerts J., Andrews T.D., Barnes C., Campbell P. (2010). Origins and functional impact of copy number variation in the human genome. Nature.

[82] Handsaker R.E., Van Doren V., Berman J.R., Genovese G., Kashin S., Boettger L.M., McCarroll S.A. (2015). Large multiallelic copy number variations in humans. Nat. Genet..

[83] Zhou B., Ho S.S., Zhang X., Pattni R., Haraksingh R.R., Urban A.E. (2018). Whole-genome sequencing analysis of CNV using low-coverage and paired-end strategies is efficient and outperforms array-based CNV analysis. J. Med Genet..

[84] Tan R., Wang Y., Kleinstein S.E., Liu Y., Zhu X., Guo H., Jiang Q., Allen A.S., Zhu M. (2014). An evaluation of copy number variation detection tools from whole-exome sequencing data. Hum. Mutat..

[85] Liu B., Morrison C.D., Johnson C.S., Trump D.L., Qin M., Conroy J.C., Wang J., Liu S. (2013). Computational methods for detecting copy number variations in cancer genome using next generation sequencing: Principles and challenges. Oncotarget.

[86] Abyzov A., Urban A.E., Snyder M., Gerstein M. (2011). CNVnator: An approach to discover, genotype, and characterize typical and atypical CNVs from family and population genome sequencing. Genome Res..

[87] Xie C., Tammi M.T. (2009). CNV-seq, a new method to detect copy number variation using high-throughput sequencing. BMC Bioinform..

[88] Krumm N., Sudmant P.H., Ko A., O’Roak B.J., Malig M., Coe B.P., Project N.E.S., Quinlan A.R., Nickerson D.A., Eichler E.E. (2012). Copy number variation detection and genotyping from exome sequence data. Genome Res..

[89] Boeva V., Popova T., Bleakley K., Chiche P., Cappo J., Schleiermacher G., Janoueix-Lerosey I., Delattre O., Barillot E. (2012). Control-FREEC: A tool for assessing copy number and allelic content using next-generation sequencing data. Bioinformatics.

[90] Sathirapongsasuti J.F., Lee H., Horst B.A., Brunner G., Cochran A.J., Binder S., Quackenbush J., Nelson S.F. (2011). Exome sequencing-based copy-number variation and loss of heterozygosity detection: ExomeCNV. Bioinformatics.

[91] Fromer M., Moran J.L., Chambert K., Banks E., Bergen S.E., Ruderfer D.M., Handsaker R.E., McCarroll S.A., O’Donovan M.C., Owen M.J. (2012). Discovery and statistical genotyping of copy-number variation from whole-exome sequencing depth. Am. J. Hum. Genet..

[92] Plagnol V., Curtis J., Epstein M., Mok K.Y., Stebbings E., Grigoriadou S., Wood N.W., Hambleton S., Burns S.O., Thrasher A.J. (2012). A robust model for read count data in exome sequencing experiments and implications for copy number variant calling. Bioinformatics.

[93] Klambauer G., Schwarzbauer K., Mayr A., Clevert D.A., Mitterecker A., Bodenhofer U., Hochreiter S. (2012). cn.MOPS: Mixture of Poissons for discovering copy number variations in next-generation sequencing data with a low false discovery rate. Nucleic Acids Res..

[94] Talevich E., Shain A.H., Botton T., Bastian B.C. (2016). CNVkit: Genome-Wide Copy Number Detection and Visualization from Targeted DNA Sequencing. PLoS Comput. Biol..

[95] Li J., Lupat R., Amarasinghe K.C., Thompson E.R., Doyle M.A., Ryland G.L., Tothill R.W., Halgamuge S.K., Campbell I.G., Gorringe K.L. (2012). CONTRA: Copy number analysis for targeted resequencing. Bioinformatics.

[96] Favero F., Joshi T., Marquard A.M., Birkbak N.J., Krzystanek M., Li Q., Szallasi Z., Eklund A.C. (2015). Sequenza: Allele-specific copy number and mutation profiles from tumor sequencing data. Ann. Oncol. Off. J. Eur. Soc. Med Oncol..

[97] Magi A., Tattini L., Cifola I., D’Aurizio R., Benelli M., Mangano E., Battaglia C., Bonora E., Kurg A., Seri M. (2013). EXCAVATOR: Detecting copy number variants from whole-exome sequencing data. Genome Biol..

[98] Jiang Y., Oldridge D.A., Diskin S.J., Zhang N.R. (2015). CODEX: A normalization and copy number variation detection method for whole exome sequencing. Nucleic Acids Res..

[99] Amarasinghe K.C., Li J., Hunter S.M., Ryland G.L., Cowin P.A., Campbell I.G., Halgamuge S.K. (2014). Inferring copy number and genotype in tumour exome data. BMC Genom..

[100] Deng X. (2011). SeqGene: A comprehensive software solution for mining exome- and transcriptome- sequencing data. BMC Bioinform..

[101] Shi Y., Majewski J. (2013). FishingCNV: A graphical software package for detecting rare copy number variations in exome-sequencing data. Bioinformatics.

[102] Gambin T., Akdemir Z.C., Yuan B., Gu S., Chiang T., Carvalho C.M.B., Shaw C., Jhangiani S., Boone P.M., Eldomery M.K. (2017). Homozygous and hemizygous CNV detection from exome sequencing data in a Mendelian disease cohort. Nucleic Acids Res..

[103] Coin L.J., Cao D., Ren J., Zuo X., Sun L., Yang S., Zhang X., Cui Y., Li Y., Jin X. (2012). An exome sequencing pipeline for identifying and genotyping common CNVs associated with disease with application to psoriasis. Bioinformatics.

[104] Packer J.S., Maxwell E.K., O’Dushlaine C., Lopez A.E., Dewey F.E., Chernomorsky R., Baras A., Overton J.D., Habegger L., Reid J.G. (2016). CLAMMS: A scalable algorithm for calling common and rare copy number variants from exome sequencing data. Bioinformatics.

[105] Chen H., Bell J.M., Zavala N.A., Ji H.P., Zhang N.R. (2015). Allele-specific copy number profiling by next-generation DNA sequencing. Nucleic Acids Res..

[106] Zhang Z., Hao K. (2015). SAAS-CNV: A Joint Segmentation Approach on Aggregated and Allele Specific Signals for the Identification of Somatic Copy Number Alterations with Next-Generation Sequencing Data. PLoS Comput. Biol..

[107] Straver R., Weiss M.M., Waisfisz Q., Sistermans E.A., Reinders M.J.T. (2017). WISExome: A within-sample comparison approach to detect copy number variations in whole exome sequencing data. Eur. J. Hum. Genet..

[108] Zare F., Dow M., Monteleone N., Hosny A., Nabavi S. (2017). An evaluation of copy number variation detection tools for cancer using whole exome sequencing data. BMC Bioinform..

[109] Kim H.Y., Choi J.W., Lee J.Y., Kong G. (2017). Gene-based comparative analysis of tools for estimating copy number alterations using whole-exome sequencing data. Oncotarget.

[110] Alkodsi A., Louhimo R., Hautaniemi S. (2015). Comparative analysis of methods for identifying somatic copy number alterations from deep sequencing data. Brief. Bioinform..

[111] Nam J.Y., Kim N.K., Kim S.C., Joung J.G., Xi R., Lee S., Park P.J., Park W.Y. (2016). Evaluation of somatic copy number estimation tools for whole-exome sequencing data. Brief. Bioinform..

[112] Gao J., Wan C., Zhang H., Li A., Zang Q., Ban R., Ali A., Yu Z., Shi Q., Jiang X. (2017). Anaconda: AN automated pipeline for somatic COpy Number variation Detection and Annotation from tumor exome sequencing data. BMC Bioinform..

[113] Jiang Y., Wang R., Urrutia E., Anastopoulos I.N., Nathanson K.L., Zhang N.R. (2018). CODEX2: Full-spectrum copy number variation detection by high-throughput DNA sequencing. Genome Biol..

[114] Chatterjee N., Walker G.C. (2017). Mechanisms of DNA damage, repair, and mutagenesis. Environ. Mol. Mutagen..

[115] Shee C., Gibson J.L., Rosenberg S.M. (2012). Two mechanisms produce mutation hotspots at DNA breaks in Escherichia coli. Cell Rep..

[116] Hoppe M.M., Sundar R., Tan D.S.P., Jeyasekharan A.D. (2018). Biomarkers for Homologous Recombination Deficiency in Cancer. J. Natl. Cancer Inst..

[117] Davies H., Glodzik D., Morganella S., Yates L.R., Staaf J., Zou X., Ramakrishna M., Martin S., Boyault S., Sieuwerts A.M. (2017). HRDetect is a predictor of BRCA1 and BRCA2 deficiency based on mutational signatures. Nat. Med..

[118] Sztupinszki Z., Diossy M., Krzystanek M., Reiniger L., Csabai I., Favero F., Birkbak N.J., Eklund A.C., Syed A., Szallasi Z. (2018). Migrating the SNP array-based homologous recombination deficiency measures to next generation sequencing data of breast cancer. NPJ Breast Cancer.

[119] Riaz N., Blecua P., Lim R.S., Shen R., Higginson D.S., Weinhold N., Norton L., Weigelt B., Powell S.N., Reis-Filho J.S. (2017). Pan-cancer analysis of bi-allelic alterations in homologous recombination DNA repair genes. Nat. Commun..

[120] Hargadon K.M., Johnson C.E., Williams C.J. (2018). Immune checkpoint blockade therapy for cancer: An overview of FDA-approved immune checkpoint inhibitors. Int. Immunopharmacol..

[121] Melendez B., Van Campenhout C., Rorive S., Remmelink M., Salmon I., D’Haene N. (2018). Methods of measurement for tumor mutational burden in tumor tissue. Transl. Lung Cancer Res..

[122] Buttner R., Longshore J.W., Lopez-Rios F., Merkelbach-Bruse S., Normanno N., Rouleau E., Penault-Llorca F. (2019). Implementing TMB measurement in clinical practice: Considerations on assay requirements. ESMO Open.

[123] FoCR (2018). Friends of Cancer Research Announces Launch of Phase II TMB Harmonization Project.

[124] Alexandrov L.B., Nik-Zainal S., Wedge D.C., Aparicio S.A., Behjati S., Biankin A.V., Bignell G.R., Bolli N., Borg A., Borresen-Dale A.L. (2013). Signatures of mutational processes in human cancer. Nature.

[125] Alexandrov L.B., Kim J., Haradhvala N.J., Huang M.N., Ng A.W., Wu Y., Boot A., Covington K.R., Gordenin D.A., Bergstrom E.N. (2019). The Repertoire of Mutational Signatures in Human Cancer. bioRxiv.

[126] Rosenthal R., McGranahan N., Herrero J., Taylor B.S., Swanton C. (2016). DeconstructSigs: Delineating mutational processes in single tumors distinguishes DNA repair deficiencies and patterns of carcinoma evolution. Genome Biol..

[127] Blokzijl F., Janssen R., van Boxtel R., Cuppen E. (2018). MutationalPatterns: Comprehensive genome-wide analysis of mutational processes. Genome Med..

[128] Maura F., Degasperi A., Nadeu F., Leongamornlert D., Davies H., Moore L., Royo R., Ziccheddu B., Puente X.S., Avet-Loiseau H. (2019). A practical guide for mutational signature analysis in hematological malignancies. Nat. Commun..

[129] Van Hoeck A., Tjoonk N.H., van Boxtel R., Cuppen E. (2019). Portrait of a cancer: Mutational signature analyses for cancer diagnostics. BMC Cancer.

[130] Germano G., Lamba S., Rospo G., Barault L., Magri A., Maione F., Russo M., Crisafulli G., Bartolini A., Lerda G. (2017). Inactivation of DNA repair triggers neoantigen generation and impairs tumour growth. Nature.

[131] Baudrin L.G., Deleuze J.F., How-Kit A. (2018). Molecular and Computational Methods for the Detection of Microsatellite Instability in Cancer. Front. Oncol..

[132] Kautto E.A., Bonneville R., Miya J., Yu L., Krook M.A., Reeser J.W., Roychowdhury S. (2017). Performance evaluation for rapid detection of pan-cancer microsatellite instability with MANTIS. Oncotarget.

[133] Huang M.N., McPherson J.R., Cutcutache I., Teh B.T., Tan P., Rozen S.G. (2015). MSIseq: Software for Assessing Microsatellite Instability from Catalogs of Somatic Mutations. Sci. Rep..

[134] Wang C., Liang C. (2018). MSIpred: A python package for tumor microsatellite instability classification from tumor mutation annotation data using a support vector machine. Sci. Rep..

[135] Foltz S.M., Liang W.W., Xie M., Ding L. (2017). MIRMMR: Binary classification of microsatellite instability using methylation and mutations. Bioinformatics.

[136] Hause R.J., Pritchard C.C., Shendure J., Salipante S.J. (2016). Classification and characterization of microsatellite instability across 18 cancer types. Nat. Med..

[137] Xia J., Chiu L.Y., Nehring R.B., Bravo Nunez M.A., Mei Q., Perez M., Zhai Y., Fitzgerald D.M., Pribis J.P., Wang Y. (2019). Bacteria-to-Human Protein Networks Reveal Origins of Endogenous DNA Damage. Cell.

[138] Santarpia L., Bottai G., Kelly C.M., Gyorffy B., Szekely B., Pusztai L. (2016). Deciphering and Targeting Oncogenic Mutations and Pathways in Breast Cancer. Oncologist.

[139] Oh B.Y., Shin H.T., Yun J.W., Kim K.T., Kim J., Bae J.S., Cho Y.B., Lee W.Y., Yun S.H., Park Y.A. (2019). Intratumor heterogeneity inferred from targeted deep sequencing as a prognostic indicator. Sci. Rep..

[140] Goh G., McGranahan N., Wilson G.A. (2019). Computational Methods for Analysis of Tumor Clonality and Evolutionary History. Methods Mol. Biol..

[141] Miura S., Gomez K., Murillo O., Huuki L.A., Vu T., Buturla T., Kumar S. (2018). Predicting clone genotypes from tumor bulk sequencing of multiple samples. Bioinformatics.

[142] Miura S., Vu T., Deng J., Buturla T., Choi J., Kumar S. (2019). Power and pitfalls of computational methods for inferring clone phylogenies and mutation orders from bulk sequencing data. bioRxiv.

[143] Pongor L., Harami-Papp H., Mehes E., Czirok A., Gyorffy B. (2017). Cell Dispersal Influences Tumor Heterogeneity and Introduces a Bias in NGS Data Interpretation. Sci. Rep..

[144] Yang L., Lee M.S., Lu H., Oh D.Y., Kim Y.J., Park D., Park G., Ren X., Bristow C.A., Haseley P.S. (2016). Analyzing Somatic Genome Rearrangements in Human Cancers by Using Whole-Exome Sequencing. Am. J. Hum. Genet..

[145] D’Agaro E. (2018). Artificial intelligence used in genome analysis studies. EuroBiotech J..

